# A Cost-Effective Transparency-Based Digital Imaging for Efficient and Accurate Wound Area Measurement

**DOI:** 10.1371/journal.pone.0038069

**Published:** 2012-05-30

**Authors:** Pei-Nan Li, Hong Li, Mo-Li Wu, Shou-Yu Wang, Qing-You Kong, Zhen Zhang, Yuan Sun, Jia Liu, De-Cheng Lv

**Affiliations:** 1 Department of Orthopedic Surgery, First Clinical College, Dalian Medical University, Dalian, China; 2 Department of Cell Biology, College of Medical Sciences, Dalian Medical University, Dalian, China; Medical College of Georgia, United States of America

## Abstract

Wound measurement is an objective and direct way to trace the course of wound healing and to evaluate therapeutic efficacy. Nevertheless, the accuracy and efficiency of the current measurement methods need to be improved. Taking the advantages of reliability of transparency tracing and the accuracy of computer-aided digital imaging, a transparency-based digital imaging approach is established, by which data from 340 wound tracing were collected from 6 experimental groups (8 rats/group) at 8 experimental time points (Day 1, 3, 5, 7, 10, 12, 14 and 16) and orderly archived onto a transparency model sheet. This sheet was scanned and its image was saved in JPG form. Since a set of standard area units from 1 mm^2^ to 1 cm^2^ was integrated into the sheet, the tracing areas in JPG image were measured directly, using the “Magnetic lasso tool” in Adobe Photoshop program. The pixel values/PVs of individual outlined regions were obtained and recorded in an average speed of 27 second/region. All PV data were saved in an excel form and their corresponding areas were calculated simultaneously by the formula of Y (PV of the outlined region)/X (PV of standard area unit) × Z (area of standard unit). It took a researcher less than 3 hours to finish area calculation of 340 regions. In contrast, over 3 hours were expended by three skillful researchers to accomplish the above work with traditional transparency-based method. Moreover, unlike the results obtained traditionally, little variation was found among the data calculated by different persons and the standard area units in different sizes and shapes. Given its accurate, reproductive and efficient properties, this transparency-based digital imaging approach would be of significant values in basic wound healing research and clinical practice.

## Introduction

Measurement of wound area is a generally objective and straightforward method to trace the course of wound healing and to evaluate the therapeutic outcome. Multiple measurement methods have been developed and used in clinical practice and experimental studies [1]. Among those methods, tracings of the healing wound with transparent acetate film [2] or by digital photograph such as stereophotogrammetry/SPG [3], [4] are the commonly employed ones. Conventionally, the wound area is defined on the transparency films, cut off along the traced margin, weighed on an accurate balancer and the wound region was estimated using the weight of a basic area unit as control [2]. Alternatively, the individual wound can be recorded in intervals by photograph and then saved in computer. The wound area is then calculated by adjusting the image of the metric ruler matched to the ruler used in the original photograph [4], [5].

It is clear that the above methods can trace the progression of wound healing simply and economically and that the transparency approach is the direct way to outline the wound margin. However, sufficient time and, sometimes, several persons are required to finish the measurement works, especially when dozens of tracing data from multiple experimental groups should be analyzed together. Besides, multiple steps of manual performance or adjustment may reduce the reliability, resulting in calculation bias [3], [6]. Apparently, a more accurate and efficient method for wound healing measurement would be necessary in clinical and translational research [7], [8]. To reach this goal, we have developed a transparency-based digital imaging approach by the use of rat skin wound model. The reliability and efficiency of this method is compared with that of traditional transparency-based weight counting approach [2].

## Methods

### Wounding and Treatment

**Figure 1:**
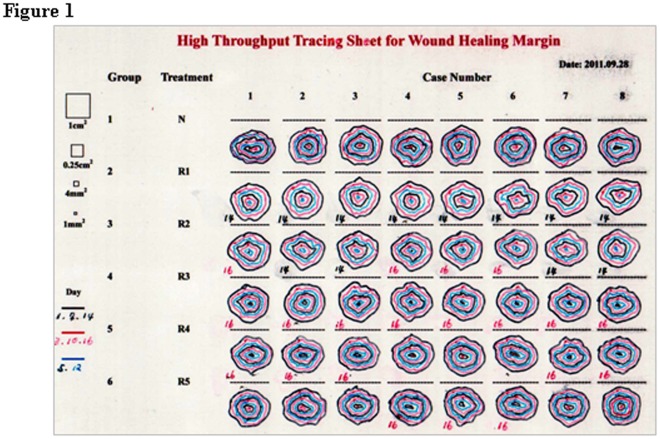
A transparency model sheet was designed in this study for wound area assessment, which included 340 pieces of tracing results, the date of experiment, observation times, the types of treatments, group numbers, animal/sample numbers, the time of wound closure as well as a set of standard area units/controls.

The desigon of the current study was carefully reviewed and specifically approved by Institutional Ethics Committee and the Committee on Research Animal Care of Dalian Medical University. After getting the permission to conduct the animal experiment, 48 five-week old male Wistar rats were provided by the Experimental Animal Center of Dalian Medical University and reared under specific pathogen-free/SPF condition. The rats were anaesthetized with 12 mg/kg xylazine via intraperitoneal injection and their dorsal surfaces were shaved and *Φ*1.5 cm^2^ round open wounds down to the muscle fascia were made on the left flank by removing the skin layer (epidermis and dermis) [9]. The animals were divided into 6 experimental groups in 8 animals/group as N, vaseline treatment as untreated control; R1 to R5, treated by a mixture of vaseline and maggot extract in 5 concentrations, respectively. The treatments lasted for 16 days by daily dressing the reagents. The margins of individual wounds were outlined at day 1, day 3, day 5, day 7, day 10, day 12, day 14 and day 16 by directly placing a transparency model sheet on the wounds. Finally, 340 pieces of tracing message were collected from 48 open skin wounds during the experiment (Figure 1).

### Design of Model Sheet and Message Transfer

A high efficient transparency model sheet for wound area assessment was designed, in which the date of experiment/observation, the types of treatments, group numbers, animal/case numbers and a set of square standard area units/intrinsic references were included (Figure 1). At each tracing time point, the same model sheet was placed over individual wounds one by one and the wound margins were marked out at the given positions (Figure 1). The colors of tracing lines were changed to easily distinguish the observation times. By the end of the experiment, the tracing data were transferred to computer by scanning the model sheet with HP Laser Jet M1005MFP and the image was directly saved in a personal computer in JPG form for digital wound area measurement. Alternatively, the transparency model sheet can be photographed with a digital camera or a cell phone and then transfer the image to computer.

### Computer-aided Wound Area Measurement

**Figure 2:**
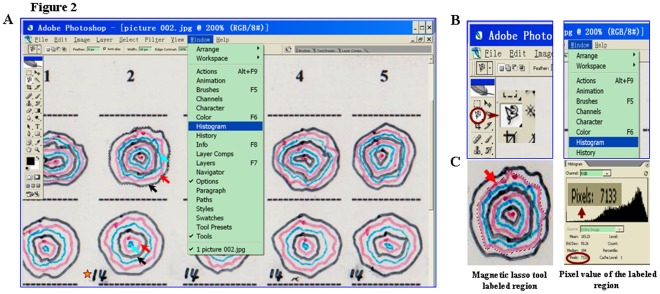
Computer-aided wound area measurement with everyday-used software. A. JPG image of the model sheet displayed in the window of Adobe Photoshop. Arrows indicate the wound regions outlined in turn in black, red and blue at Day-1, Day-3 and Day-5 and then at Day-7, Day-10 and Day-12 time points. Star mark: the day of wound healing. B. The locations of magnetic lasso tool button for defining the selected traced region (left) and “Histogram” button for calculating pixel value (right). C. Arrow indicates Magnetic lasso tool labeled R1 No. 2 wound region at Day-3. The pixel value of this region was calculated by pressing “Histogram” button.

To calculate wound area, the image of scanned JPG model sheet was opened at Adobe Photoshop CS4 site (Figure 2A). A given wound was defined along its border by pressing button of so-called “magnetic lasso tool” (Figure 2B). The pixel value of the target region was calculated by pressing “Histogram” button (Figure 2C) and then it was recorded in an excel form. This performance was repeated until the pixel values of all marked regions were counted and recorded. A standard area unit was chosen according to the overall sizes of the wounds and its pixel value was cited as numerator. The areas of all wounds were calculated simultaneously by dividing each of the wound pixel values (Y) in the excel form with the pixel value of standard area unit (X) and then times the real area of the standard unit (Z), Y/X × Z. When Z value is one, e.g. 1 mm^2^ and 1 cm^2^, the area calculation can be simply done by Y/X. The wound healing statuses of individual experimental groups at different tracing times were elucidated according to their mean values and statistical analysis (ANOVA). Calculation was conducted by three independent researchers and the data they obtained and the times they expended were compared.

### Traditional Transparency-based Wound Assessment

**Figure 3:**
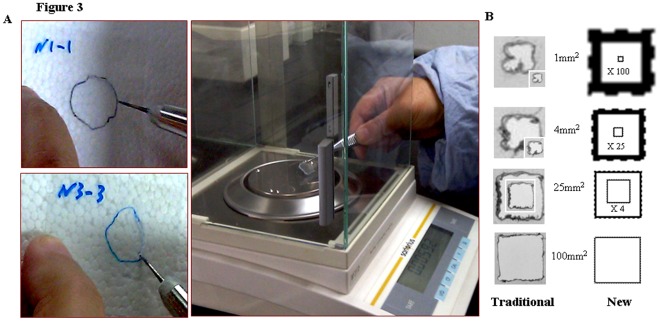
Shortcomings of traditional transparency-based wound assessment. A. In traditional transparency-based wound healing assessment, a hundred pieces of transparency films had to be prepared for marking out the margins of individual wounds at different observation times. The outlined regions as well as the suitable standard area control(s) were cut off along the margin with an electronic cutter and weighed on an analytical balancer. B. The shapes and relative sizes of four standard area units isolated by electronic cutter (Traditional) or labeled with Magnetic lasso tool in Adobe Photoshop program (New). The insets are the images in original sizes.

The traditional transparency-based wound healing assessment was cited as an efficient and accurate control. Briefly, more than a hundred pieces of transparency films were prepared for marking out the margins of individual wounds at different observation times. Under the cooperation of three researchers, the outlined regions as well as the suitable standard area control(s) were cut off along the margin with an electronic cutter (Figure 3A) and weighed by an analytical balance (Figure 3B; Sartorius, Weender Landstrasse, Goettingen, German). The weights of transparency pieces were converted to the areas one by one by dividing the weight of the checked region (Y) with the weight of standard unit (X) and then times the known area of the standard unit (Z), Y/X × Z. Because of the difficulty to exactly cut off tiny area from the transparency, 25 mm^2^ (5 mm×5 mm) had to be used as the standard unit in our case. Similarly, the wound healing statuses of each of the experimental groups were evaluated according to their mean values and statistical analysis (ANOVA).

### Comparison of Calculation Accuracy of Square and Circle Standard Units

Although the square standard units make the digital method easy to use, the wounds usually seen are elliptical or round in shape. To elucidate the suitability of square standard units in calculating the round wounds, a square (25 mm^2^) and a circle (24.62 mm^2^) standard units were prepared with a personal computer/PC, which was used directly to assess the sizes of a set of computer-designed standard round objects in known areas. Alternatively, these two units were printed to transparency film and scanned together with the irregular wound tracings to be measured by them. The above assessments were conducted for 5 times to assess the mean sizes of individual regions. The agreement between the areas measured with the square/S and the circle/C standard area units was assessed by plotting the areas in a Bland Altman Plot [10]. The coefficient of variation (CV) was generated by dividing the standard deviation (SD) of the ratio of S and C by (S + C)/2 times 100 [11].

## Results

### Cost-effective Wound Tracing, Data Conversion and Area Assessment

Since 32 wounds healed at the day 14 or day 16 of the experiment (the star mark in Figure 2A), 340 wound tracing results were collected and documented compactly in a transparency model sheet. This sheet was then scanned and the defined areas in the JPG image were directly used for area calculation because of the presence of 4 standard area units in the sheet as intrinsic references. By use of the magnetic lasso tool and “Histogram” in Adobe Photoshop software, a target wound area was defined and its pixel value was counted (Figure 2B and 2C). The magnifications of the images could be adjusted without the pixel re-judgment of standard area units. Since the above performance could be finished within 27 seconds (Table 1), it took the three researchers 142, 150 and 152 minutes respectively to accomplish area assessments for 340 regions. In contrast, the three researchers spent 198 minutes (35 seconds/tracing) to finish the same task with the traditional transparency method (Table 1). Moreover, the traditional performance became especially difficult when working on small/tiny tracing data or area units such as 1 mm^2^, leading to remarkable variations of area assessment (Figure 3).

**Table 1:** Average wound areas of two experimental groups calculated with two methods on Day-10 for three times.

Method	Average wound areas (mm^2^; X±S) ^$^			Time used (seconds; X±S)		
	1	2	3	1	2	3
**New**						
N*	29.24±3.10	29.62±3.56	30.1±3.35	23.38±3.16	25.63±4.03	25±3.21
R1*	7.24±0.96	7.16±1.31	7.37±1.27	23.13±2.03	24.38±1.92	23.25±2.43
**Traditional**						
N*	19.46±1.87	19.10±2.28	19.99±2.03	38.71±3.20	40.75±1.83	39.37±3.46
R1^#^	5.09±1.07	6.19±0.80	4.77±0.97	36.25±1.98	35.13±4.12	38±1.85

$, the value and the weight of 1 mm^2^ standard unit were cited as area control. * *P*>0.05; ^#^, *P*<0.01.

### Remarkable Reduction of Calculation Bias

**Figure 4:**
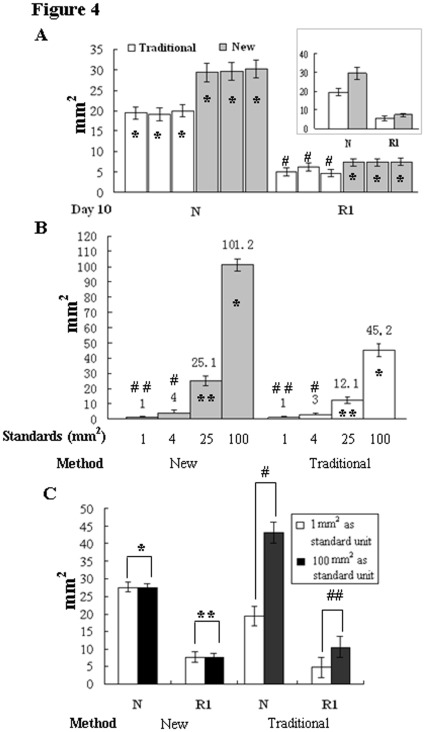
Remarkable differences of measurement accuracy between transparency-based digital imaging and traditional transparency methods. A. The wound areas of non-treated (N) and Reagent-1 treated (R1) groups calculated with transparency-based digital imaging (New) by three researchers and with transparency tracing method (Traditional) for three times on Day-10. Upper-right inset: the average sizes of the calculated regions with the two methods. B. Comparison of the accuracy of the calculated area data generated from transparency-based digital imaging and traditional transparency methods. The numbers on the top of each of the columns indicate the areas calculated according the pixel values or the transparency weights of the four standard units (1 mm^2^, 4 mm^2^, 25 mm^2^ and 100 mm^2^). *, *P* = 0.000<0.01; **, *P* = 0.000<0.01. ^#^, *P*  = 0.081>0.05; ^##^, *P* = 0.979>0.05. C. Reproducibility of the new and traditional wound assessment methods performed on Day-10 traced data. The pixel values and the transparency weights of 1 mm^2^ and 100 mm^2^ standard units were used as numerators, respectively. ^*^, *P* = 0.857>0.05; ^**^, *P* = 0.889>0.05. ^#^, *P* = 0.000<0.01; ^##^, *P* = 0.000<0.01.

Three persons including a graduate medical student measured the areas of wound regions independently, using the same JPG image and calculation protocol. Comparing their results, the calculated areas of individual wound sites and the mean area values of each of the experimental groups were almost identical (ANOVA, *P*>0.05; Figure 4A and Table 1), regardless of the types of standard area units used (Figure 4B). In contrast, the calculated areas of the wound sites with traditional transparency method were highly variable when different area units were used (Figure 4B and 4C; *P<0.05*). Furthermore, pixel values rather than the weights of isolated transparencies of the four standard area units were well matched with each other (Figure 4B). Because of the difficulty in isolating the target regions precisely (Figure 3B), the areas calculated traditionally were smaller than that based on pixel values (Figure 4B), which was largely due to the weight loss of area units during electronic cutting along the tracing margins.

### Accurate Measurement with both Square and Circle Standard Units

**Figure 5:**
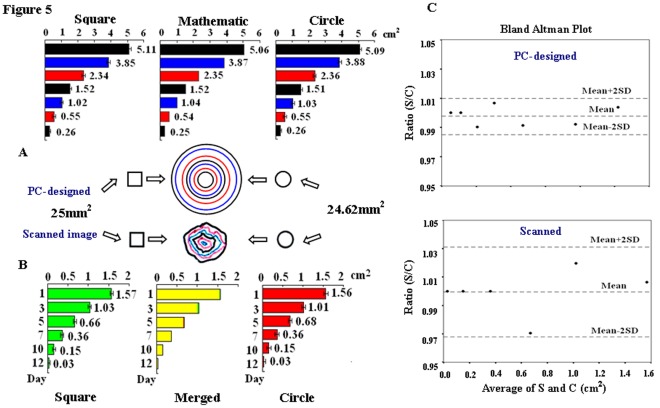
Comparison of the areas calculated with a square (25 mm^2^) and a circle (24.62 mm^2^) standard units. A. Area calculation performed on seven personal computer/PC-designed round standard controls in the known mathematic sizes. No statistical difference was found between the mathematic areas and the areas calculated with the PC-designed square or circle area unit (*t*-test; *P*>0.05). B. Area calculation performed on six transparency tracings marked out from a wound. In the middle histogram, the yellow part of the column indicates the merged areas calculated with the square (red) and the circle (green) standard unit. No distinct variation was found between the areas calculated with the scanned square and circle standard area units (*t*-test; *P*>0.05). C. Bland Altman Plots for assessing agreement between the areas measured with 25 mm^2^ square (S) and 24.62 mm^2^ circle (C) standard units. Mean±2SD represents 95% confidence interval of the Ratio (S/C). Coefficient of variation (CV): 0.31% for PC-designed and 2.52% for scanned objects. 95% limits of agreement: 0.98-1.01 for PC-designed and 0.97-1.03 for scanned objects.

In this experiment, the areas of a set of PC-designed standard round objects in the known mathematic areas were calculated by a 25 mm^2^ square and a 24.62 mm^2^ circle standard units, respectively. Meanwhile, the scanned square and circle standard units in the same sizes were used to measure the irregular wound tracings in JPG image. It was shown that in the former case, the areas calculated with both units were largely identical (*P*>0.05) and well matched with the mathematic areas of the objects (Figure 5A); in the later case, the pixel values and the calculated areas generated from the two types of standard area units were almost overlapped (Figure 5B). According to the results of Bland Altman Plot, the areas measured with the square and the circle standard units were in good agreement in both PC-designed and scanned cases (Figure 5C). The results also revealed that to assess the real areas of the tracings, the standard area controls and the samples to be measured should be prepared under the same conditions (data not shown).

## Discussion

Multiple approaches for wound assessment have been developed and used in clinical practice and basic research [12], [13], including the traditional tracing of wound margin onto transparent acetate film [1], [2] and the later developed computer-aided digital imaging method [3], [8]. Nevertheless, the accuracy and efficiency of the above assessments needs to be improved especially when a large amount of data needs to be processed. Alternatively, the easy-performance of transparency tracing [1], [14] and the accuracy of digital imaging [3] lead us to combine them for more efficient and precise measurement of skin wound healing.

As the precedent condition to improve current wound healing assessment methodology, a cost-effective transparency model sheet was designed in which dozens to hundreds of tracing data could be documented in well arranged and highly compact manner. As presented in the current study, the skin wound margins of 6 experimental groups (8 rats/group) were traced in 8 observation intervals, generating 340 pieces of tracing results on the model sheet. Since the wound sizes depict progressive reduction, the margins of the same wound traced over time are integrated at a given position. By this way, hundreds of tracing results can be accommodated in one model sheet and the area measurement of individual wounds is conducted more handily. In case a wound repairs slowly, the wound margins are outlined regularly in different colors such as black, red and then blue to distinguish the tracing times clearly. Furthermore, the style of model sheet can be modified by researchers according to their research designs, and the standard area controls in different sizes and shapes such as square and round can be use equivalently.

Tracing of the wound margin onto transparency film is a simple and economic way for measuring the wound areas [15], [16]. However, it is beyond its reach when a large amount of tracing data should be documented, calculated and analyzed together. Additionally, the measurements of the tracing area on transparency film by weighing the outlined region or measuring the wound diameter or matching the tracing to a known area are far more exact, especially when the wounds are in irregular sizes or too small to be precisely isolated. According to our experience, even in case of well controlled animal model, the wounds of the skin keep changing shapes as times. This shortcoming of transparency-based method has been overcome here by scanning the data-containing model sheet and saving the scanned image in JPG form. More importantly, the image can be directly used for measuring the wound areas without any adjustment because of pre-integration of a set of standard area units in the model sheet.

Several accurate digital image-based wound area measurement methods have been described but they are usually employed to calculate the separate tracing images [3], [17] rather than a vast amount of data highly packaged into a single image as shown in current study. Besides, the manners to measure the tracing area are different because special software and/or instruments are required [13], [18]–[21]. As described in the sections of Methods and Results, the “magnetic lasso tool” in everyday-used Adobe Photoshop program was adopted by which the pixel value of the exactly defined region could be obtained automatically, which was then divided by the pixel value of a known area to get the true size. In this study, we scanned the transparency model sheet containing over three hundred tracing data to JPG image, followed by counting the pixel values one by one and finally calculating the areas of individual outlined regions together. By this way, less than 3 hours would be sufficient for a researcher to finish the total calculation works. In contrast, over 3 hours were required by three researchers to jointly accomplish the same task using the traditional transparency method. The accuracy of this approach is also outstanding in terms of minimized variation of the calculated data among different performers and perfectly matched pixel values of 4 standard area controls. As shown in Table 1 and Figure 3A, the average sizes of skin wounds of untreated group and the animals treated with Reagent-1 at Day 10 were calculated by the three persons and showed little deviation (*P* = 0.079>0.05; *P* = 0.318>0.05). When the conventional transparency method was used, apparent bias (*P* = 0.018<0.05 to *P* = 0.687>0.05) was found in the results obtained by repeated calculation of the outlined regions copied from the same transparency films. The well matched pixel values among the four square standard area units and, especially, good agreement of the areas estimated by the round and the square units further demonstrated that in addition to the high efficiency and cost-effectiveness, our novel method is more precise and reproducible in comparison with the wound area assessment methods so far available.

Taken together, an efficient, accurate and practical approach for wound healing assessment is established in current study by combining the advantages of transparency tracing and digital imaging. Multiple analyses demonstrate that this method permits us to conduct comprehensive wound healing studies in more precise and cost-effective manner. Since hundreds of wound areas can be documented simply and measured quickly with this novel technique, there should be no problem to handle the skin wound measurement works commonly encountered in the laboratories and clinics.
